# All Plant Breeding Technologies Are Equal, but Some Are More Equal Than Others: The Case of GM and Mutagenesis

**DOI:** 10.3389/fpls.2021.657133

**Published:** 2021-07-02

**Authors:** Luisa Batalha, Francesco Foroni, Brian Joseph Jones

**Affiliations:** ^1^School of Behavioural and Health Sciences, Australian Catholic University, Sydney, NSW, Australia; ^2^School of Life and Environmental Sciences, University of Sydney, Sydney, NSW, Australia

**Keywords:** GM, gene editing, framing, food security, mutagenesis, CRISPR, attitudes

## Abstract

A pervasive opposition to genetically modified (GM) foods has developed from the notion that they pose a risk to human and environmental health. Other techniques for the genetic modification of plants, such as sexual crossing and mutagenesis breeding, have mostly remained unchallenged. This research aims to investigate public perception of plant breeding technologies. Specifically, sexual crossing, mutagenesis, transgenics (GM) and gene editing. It was expected that attitudes and intentions would be most positive and the perception of risk lowest for plant genetic modification through sexual crosses. Scores on these variables were expected to be similar between mutagenesis, GM and gene editing. It was also expected that attitudes, intentions and risk perception would change (becoming more positive) once participants learned about foods developed through these technologies. Participants reported their attitudes, intentions and risk perception at two points in time. At Time 2, they were presented with pictures of food items developed through sexual crossing, GM and mutagenesis. The results showed that mutagenesis stood out as the most negatively perceived technology, whereas genetic development via sexual crosses was generally perceived as positive. The results highlight the importance of messaging, framing in consumer attitudes.

## Introduction

Humans have been purposefully modifying the genetics of plants to improve traits since at least the early 20th century. The methods that are now available for directly modifying the genetics of plants range from the various manifestations of traditional sexual cross-breeding through to the newly developed technique of gene editing. In this work, we discuss genetic modification in the broad sense, but use the term genetic modification (GM) specifically to mean laboratory-based transgenic techniques. Almost since their inception, GM food crops have attracted controversy and consumer resistance. Whilst scientists have argued that there is no more danger in GM methods than in traditional breeding (Agre et al., 2016; Roberts, 2018), non-governmental organisations (NGOs) such as Greenpeace, contend that GM products pose potential dangers and should be banned or, at least, highly regulated because “there is not an adequate scientific understanding of their impact on the environment and human health” (Greenpeace, n.d.). Similar arguments have been mounted against the new CRISPR gene-editing technology that can induce precision targeted DNA mutations in plants and other organisms (Jones, 2015; Cotter and Perls, 2018). However, over 4,400 risk assessments have been undertaken by governments in over 70 countries, all concluding that GM crops pose risks no greater than the risks of conventional, non-GM crops (International Service for the Acquisition of Agro-Biotech Application, 2019). Thus, although the balance of scientific evidence does not support a claim that GM or gene editing techniques, or the foods derived from them are dangerous (European Commission, 2001; Panchin and Tuzhikov, 2017), an implied potential association between GM foods and environmental and human health risk is pervasive (Pham and Mandel, 2019), albeit changing in recent times (see Ichim, 2021). As a result of the concerns, the use of GM technology in plant breeding remains highly regulated (Bonny, 2003; European Commission, 2018). At the same time, techniques such as chemical or radiation-induced DNA mutagenesis have remained mostly unchallenged by NGOs and consumers. As all of these techniques (i.e., gene editing, mutagenesis, GM) have broad support within the scientific community (Savadori et al., 2004; Agre et al., 2016; Fernbach et al., 2019), the basis of the variability in consumer attitudes and regulatory controls is unclear. One key for understanding the difference between expert position and consumer attitudes might relate to how consumer attitudes are formed and/or modified. The aim of this study is to gauge public knowledge, attitudes and risk perception toward a variety of plant breeding techniques. A greater understanding of these factors could help in the development of communication strategies and decision-making related to the deployment of plant breeding technologies, both new and old.

### Plant Breeding: When Precision Means “Risk”

Humans have been selecting desirable traits in plants since the beginning of agriculture (Wieczorek and Wright, 2012). Over time, selection and the targeted breeding that has followed it have resulted in marked changes in the genetics of agricultural plants. The morphological and physiological advantages that current agricultural plants have, compared to their wild type ancestors, flow from these genetic changes (Luckett and Halloran, 2017). Modern plant breeders combine DNA from whole genomes to short, specific DNA sequences originating from both closely and distantly related species, in order to improve plant characteristics and performance. They are also able to create novel traits by inducing DNA mutations *de novo*. The currently used plant breeding techniques fall along a continuum of precision, from the relative imprecision of combining whole genomes through sexual crossing, through to the introduction of random mutations through chemical or radiation induced mutagenesis, and to precise, small-scale changes through techniques such as GM or gene-editing (see Appendix B for a description of each technology). Although, the risk (or lack thereof) of environmental or human health impacts associated with these techniques are arguably similar, GM technology has been categorized by some people as inherently more dangerous (Trewavas and Leaver, 2001; Roberts, 2018; Williams et al., 2021).

There is a considerable body of research analyzing attitudes toward GM and genetically modified organisms (GMOs; e.g., Napier et al., 2004; Aerni and Bernauer, 2006; Costa-Font and Gil, 2009; Yamaguchi, 2013; Delwaide et al., 2015; Hudson et al., 2015; Lucht, 2015; Vecchione et al., 2015; Rzymski and Królczyk, 2016), but to the best of our knowledge there is no research investigating attitudes to other long established plant breeding technologies such as induced mutagenesis. This is the more striking as induced mutagenesis techniques have been used since the 1930s, and over 3000 induced mutant plant varieties have been registered and marketed, including commodity crops, fruit and vegetables (International Atomic Energy Agency, n.d.).

Some research has been conducted into attitudes toward the use of radiation in relation to foods. It has been found, for example, that prawns that had been made infertile through radiation induced mutagenesis were perceived negatively and similarly to GM foods in terms of health, natural content, and ecological welfare (Evans and Cox, 2006). Negative attitudes have also been found in relation to other associations between food and irradiation, such as the use of radiation for postharvest shelf-life extension (e.g., Behrens et al., 2009; Caputo, 2020). While consumer attitudes to this direct use of radiation on food products is generally negative, Caputo (2020) found that participants were more positive to food irradiation when they were presented with information that related this technology to food safety, and more favorable still when positive information about the technique was given to them. This suggests that the way information about food is framed makes a difference to the acceptance of a product.

As new breeding technologies (e.g., CRISPR) emerge, it is important to understand what the public know and think of a broad range of plant breeding technologies so that strategies can be developed that promote consistent and coherent community responses and regulatory environments.

### Framing Effects, Attitude Formation, and Change

Framing can be defined “as the process by which a source […] defines the essential problem underlying a particular social or political issue, and outlines a set of considerations purportedly relevant to that issue” (Nelson et al., 1997, p. 222). The way issues are framed has consequences for how recipients perceive the message. For example, there is evidence from survey research that when the term *biotechnology* is used attitudes are more positive than when *genetic engineering* is used (Eurobarometer, 1993). Druckman (2001) distinguishes between two types of framing effects. *Equivalency framing effect*, referring to the use of different, but equivalent words or phrases (e.g., 2% sugar vs. 98% sugar-free), where one form is aimed to induce a positive evaluation and the other a negative one. When someone reads that a food item contains 2% sugar, they may conclude that this is a substantial amount. On the other hand, reading that the same food item is 98% sugar-free, may lead the person to conclude that the food has very little sugar.

The other type is *emphasis framing effect* and refers to the emphasizing of a set of considerations to influence the recipient’s opinion. Both framing effects seem to be on display in some NGOs’ position in relation to GMOs. For example, Fairtrade International and Greenpeace argue that “the risks that GMOs pose are still unknown, and they may have unforeseeable environmental, social, and health impacts” (Cornish, 2018, n.p.). There are both negative (i.e., risks are unknown and unforeseeable) and emphasis (i.e., environmental, social, and health impacts) framing effects in this statement. This framing of GMOs in negative terms that emphasize risk on several fronts, is likely to influence the public in a predetermined direction. This works because when people are uncertain about their knowledge on an issue, they are guided by others, who they believe are more knowledgeable (i.e., informational influence; Deutsch and Gerard, 1955; Williamson et al., 2013), or believe that they have knowledge of an issue when, in reality, what they know is simply that the knowledge exists in the community (Fernbach and Sloman, 2017). There is evidence that scientific knowledge has a positive impact on public attitudes to genetic science (Sturgis et al., 2005; McPhetres et al., 2019). But, public understanding of science is low (Miller, 1998), and research shows that a lack of knowledge about science is associated with greater opposition to GM foods and stronger beliefs about one’s understanding of GM food technology (Fernbach et al., 2019). The lack of science literacy is therefore problematic as it leaves the door open to informational influence and the effects of knowledge illusion (i.e., the belief that one has knowledge when this is not true; Fernbach and Sloman, 2017). In the case of GM technology, the lack of knowledge leads the public to form opinions based on information by sources considered trustworthy, such as NGOs, who tend to be held in high regard (see GlobeScan, 2013, 2020; Hilton et al., 2013). Governments, in turn, take public opinion into account when legislating food regulations (European Commission, n.d.; Wolt and Wolf, 2018). NGOs therefore are able to influence the development of legislation and regulation both directly through lobbying, and indirectly through shaping public opinion.

The relatively recent CRISPR technology has been presented in a way similar to GM and has already faced community resistance and legislative impediments (see Cotter and Perls, 2018; Greenpeace, 2020). Interestingly, the much older mutagenesis technologies have largely escaped consumer awareness. In fact, until recently (see Conseil d’Etat/Council of the State, 2020) there have been no attempts to frame this technology within the public debate, even though research suggests that people would find mutagenesis as posing similar risks to GM (e.g., Evans and Cox, 2006).

The theory of planned behavior (Ajzen, 1991) posits that whether or not a person engages in a behavior is dependent on intention, which, in turn, is jointly dependent on one’s attitude toward the behavior, subjective norms, and perceived behavioral control. However, there are situations in which behavior does not, or cannot, follow such neat “rules” because people may not know that their behavior is in fact inconsistent with their attitudes or simply because behavior, more often than not, is not driven by reason (Kahneman, 2011). In the case of mutagenesis, consumers may have been unknowingly eating plants developed through mutagenetic processes (Caputo, 2020). It thus begs the question what consumers would do when they learn that they may have been eating foods developed through means that they deem dangerous. This knowledge is likely to posit a problem of consistency between the person’s attitude (i.e., perceiving the technology as risky) and their behavior (i.e., consuming food developed by the technology). Evidence from psychology shows that humans have a need to maintain consistency between their attitudes and behaviors (Festinger and Carlsmith, 1959). When this consistency is broken, psychological discomfort arises (i.e., cognitive dissonance), which urges the person to resolve or reduce the dissonance (Festinger, 1957). The ways of reducing a conflict between attitude and behavior are to change one or the other. Do they maintain a similar perception of risk and stop consuming that food? Or, instead, do they change their risk assessment and attitude toward that technology? The choice is limited though, if the behavior has already taken place (or is difficult to change). The only remaining alternative then, is to change one’s attitude. This suggests that negative attitudes to mutagenesis would likely change if people learned that they are already consuming foods produced through various technologies where the food is widely available and difficult to substitute (e.g., rice).

### Research Overview

The contrasting paths that mutagenesis and GM plant breeding have taken despite similar potential risks (or lack thereof), suggests that the debate is driven by something other than scientific evidence. And while scientists, governments, and NGOs drive their own agenda, the public is largely kept in the dark. This study provides information to participants about various plant breeding technologies and, in doing so, aims to frame the issues within factual scientific language. It then investigates perceptions of, and attitudes and intentions toward mutagenesis and, more pertinently, whether these perceptions, attitudes, and intentions would change once people learn that produce bred through mutagenesis is already part of their diet. The aim is to investigate people’s knowledge, attitudes, intention to consume, and risk perception in relation to various breeding technologies (i.e., sexual crossing, mutagenesis, GM, gene editing) and how factual information may change them, with the objective of examining whether there is imbalance in the nature of the debate and inconsistency in regulations.

We designed a study that aims to show that not only framing, but the terminology used in relation to food development techniques, is associated to more or less favorable attitudes toward foods developed through each breeding technique. We measured attitudes, intention to purchase, and risk perception at two time points, one week apart. We also provided participants with information sheets about each technology (at Time 1 [T1] and Time 2 [T2]) as well as examples of foods developed through sexual crossing, mutagenesis, and GM (T2 only). We did not give examples of foods developed through gene editing because such foods are not yet widely available in the market.

#### Hypotheses

Based on the reviewed literature we hypothesized that at T1:

H1a. Attitudes will be more positive for sexual crossing compared to mutagenesis, GM, and gene editing.

H1b. Attitudes will be similar among mutagenesis, GM, and gene editing.

H2a. Intentions to purchase will be stronger for sexual crossing compared to mutagenesis, GM, and gene editing.

H2b. Intentions will be similar among mutagenesis, GM, and gene editing.

H3a. Perceived risk will be lower for sexual crossing compared to mutagenesis, GM, and gene editing.

H3b. Perceived risk will be similar among mutagenesis, GM, and gene editing.

H4. We also anticipated that knowledge of breeding technologies will be generally low, and particularly low for GM and mutagenesis.

We also hypothesized that at T2, after participants learned that they were likely already eating foods developed through sexual crossing, mutagenesis and GM technologies, their scores on attitudes (i.e., more positive attitude; H5a) and intention to purchase (i.e., more likely to purchase; H5b) will increase, and those on risk perception will decrease (i.e., less risk; H5c). Due to a lack of an exemplar, no food item developed through gene-editing will be presented to participants. It is, therefore, expected that attitudes, intention to purchase, and risk perception will not change for this technology from T1 to T2 (H5d).

H6. The change from T1 to T2 in attitudes, intentions and perceived risk of sexual crossing, mutagenesis, and GM will be higher as the discrepancy between the level of consumption of foods associated with each technology and the scores for those variables increases.

We also expected that once participants had been presented with commercially available specific food items and had learned how those foods had been bred (i.e., tomatoes by sexual crossing; rice by mutagenesis; canola oil by GM), they would be similarly inclined to buy any of these food items (H7). This is because inconsistency between attitude and behavior would cause cognitive dissonance, leading to attitudes toward mutagenesis and GM foods becoming more positive.

## Method

### Participants

Because we aimed to test specific hypotheses, we decided that a convenience sample would provide reliable data. As such, the sample comprised 114 undergraduate psychology students from an Australian university (20 males, 96 females), who participated in return for course credit. Their ages ranged from 18 to 52 years (*M* = 23.62, *SD* = 6.94). We assumed that psychology students, while likely to be more educated than the general population, due to their subject matter, their knowledge of plant biotechnology would be similar to that of the lay general public and that they would be capable of understanding complex information such as that related to plant biotechnology. A research proposal addressing ethical issues related to human research was submitted and approved by the home institution’s Human Research Ethics Committee^1^.

### Design

The research was conducted as a longitudinal study with data collected at two points in time, one week apart. Baseline data collected at T1 served to test hypotheses H1–H3, as well as a basis for comparison with data collected at T2, when participants learned about foods bred through different techniques. This study was part of a larger research project and only the variables of interest are reported here.

### Measures

Unless stated otherwise, all questions in the following measures were measured on a 7-point Likert scale from 1 (*Strongly disagree*) to 7 (*Strongly agree*). Full scales for the three measures presented below can be seen in Appendix A.

#### Attitudes to Biotechnology

Attitudes to each biotechnology was measured with three items adapted from Costa-Font and Gil (2009). An example is “Food produced with this technology will be useful for the fight against third world hunger.” The internal consistency of the measure, denoted by the Cronbach’s alpha (α), suggests good reliability both at T1 (sexual crossing, α = 0.78; mutagenesis, α = 0.72; GMO, α = 0.75; CRISPR, α = 0.77) and T2 (sexual crossing, α = 0.80; mutagenesis, α = 0.82; GMO, α = 0.82; CRISPR, α = 0.80), showing that the items measured a single construct.

#### Perceived Risk

Perceived risk of each technology was also measured with three items adapted from Costa-Font and Gil (2009). An example is “Growing crops with this technology will be harmful to the environment.” The reliabilities were good at T1 (sexual crossing, α = 0.80; mutagenesis, α = 0.84; GMO, α = 0.84; CRISPR, α = 0.86) and T2 (sexual crossing, α = 0.81; mutagenesis, α = 0.82; GMO, α = 0.87; CRISPR, α = 0.86).

#### General Consumer Intentions

Consumers’ intention to purchase products developed with each technology was also measured with three items adapted from Costa-Font and Gil (2009). An example is “I would buy food developed through this technology if it were grown in a more environmentally way.” The reliabilities were good both at T1 (sexual crossing, α = 0.78; mutagenesis, α = 0.84; GMO, α = 0.81; CRISPR, α = 0.80) and T2 (sexual crossing, α = 0.78; mutagenesis, α = 0.82; GMO, α = 0.85; CRISPR, α = 0.81).

#### Consumption and Liking for Food Items

To measure participants’ history of consumption and liking for the food items associated with each technology the questions “How much do you eat rice/tomatoes/canola oil?” and “How much do you like rice/tomatoes/canola oil?” were asked on a 5-point scale from 1 (*never/don’t like it at all*) to 5 (*every day/like it a lot*).

#### Knowledge of Technology and Likelihood of Purchasing Specific Food Items

Awareness about how each of the three foods (tomatoes, rice, canola oil) were developed, and the likelihood of purchasing the specific food developed through the technology were measured with single items: “How aware were you of the way this food has been developed?” and “How likely are you to purchase [food] developed through this technology?” These questions were measured on a 5-point scale from 1 (*not at all aware/extremely unlikely*) to 5 (*very aware/extremely likely*).

#### Attention Control Questions

To ensure that participants took the survey seriously, not just ticking a number randomly and compromising the reliability of the data, control questions (e.g., “I’ve been to the moon twice”) were interspersed throughout the survey at T1 and T2. These questions were used to exclude participants that responded inappropriately to the control questions indicating that did not properly attend to the questionnaires.

### Procedure

#### Time 1

The study was conducted online through the Qualtrics survey platform. At T1, participants started by providing consent to the study, which was followed by them generating a personal code (for matching with the data at T2), and questions pertaining to their gender and age. Following this, participants read an introductory fact sheet about plant breeding, which was followed by information about four different plant breeding technologies: sexual crossing, mutagenesis, GM, and CRISPR (adapted from information in Wikipedia and researcher knowledge, see Appendix B). Immediately after the information about each technology, participants were asked about their attitudes to that specific technology, the risk associated with the technology and their intentions to buy food developed through the technology. As such, they responded to these questions four times. Finally, to ensure that participants had paid attention to the information, six technologies were presented about which participants were asked to mark the ones they had read about.

#### Time 2

At T2, participants again provided consent, generated the personal code and reported their age and gender. Following this, they were asked how often they consume and like rice, canola oil, and tomatoes after which they were shown pictures of these produces, each accompanied by information about how these foods had been improved through mutagenesis, GM, and sexual crossing, respectively. No food was presented for CRISPR because, at present, there are no commercialized foods developed through this technology (see Appendix C). Each picture and information were followed by questions about participants’ awareness of each food item’s breeding development and how likely they were to purchase them if they were developed through the respective technology. Following this, participants were given the same information about each breeding technology as at T1 (Appendix A) and answered the same questions as at T1 regarding their attitudes, perceived risk and intentions to buy food developed through each technology. They finished by identifying which technologies had been presented to, again, check that they had been paying attention.

## Results

Before we conducted any analyses, we examined the control questions to check that participants had provided informed responses. This examination showed that seven participants had given implausible answers to questions such as “I’ve been to the moon twice.” These participants were, therefore, removed from further analyses. The raw data has been placed in an open access databank^2^.

To test the hypotheses that attitudes, intention, and risk perception for sexual crossing was different from mutagenesis, GM, and gene-editing technologies (H1a, H2a, and H3a) but similar among the last three (H1b, H2b, and H3b) at baseline, we conducted a series of repeated-measures analyses of variance (ANOVA) testing these variables at T1. All within-subjects’ analyses were subjected to the Mauchly’s test of assumption of sphericity before pairwise comparisons. Cases in which this assumption was not met, Greenhouse-Geisser corrected tests were reported.

### Attitudes at T1

**FIGURE 1:**
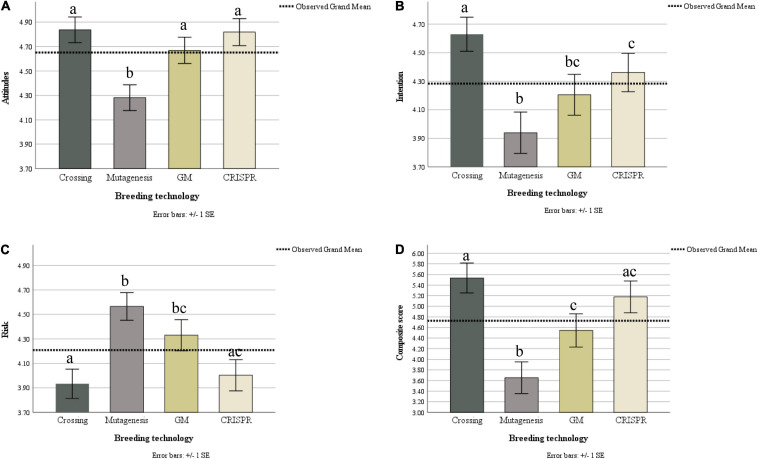
Illustration of differences between the four breeding technologies across the three variables and a composite score (all three variables combined). Different letter indicates that the means differ significantly within a panel in Bonferroni-corrected pairwise comparisons. Results in Panel **(B)** are Greenhouse-Geisser corrected. Results in panels **A**, **C**, and **D** met the assumption of sphericity.

The within-subjects test showed a large and significant effect of technology, *F*(3, 324) = 16.37, *p* < 0.001, _p_ω^2^ = 0.12. Pairwise comparisons with Bonferroni corrections show that attitudes to sexual crossing (*M* = 4.84, *SD* = 1.11) were significantly more positive than attitudes to mutagenesis (*M* = 4.28, *SD* = 1.11, *p* < 0.001), but not more positive than GM (*M* = 4.67, *SD* = 1.12, *p* = 0.356) or CRISPR (*M* = 4.82, *SD* = 1.16, *p* > 0.09). H1a was therefore only partially supported as attitudes to sexual crossing were not more positive than attitudes to all other technologies. H1b was also only partially supported as attitudes were not similar among the three other technologies. Specifically, attitudes to mutagenesis were significantly more negative than attitudes to GM (*p* = 0.001) and CRISPR (*p* < 0.001), whereas attitudes to GM and CRISPR were not statistically different (*p* = 0.446, see Figure 1A).

### Intention to Buy at T1

Within-subjects test shows a medium and significant effect of technology, *F*(2.70, 291.15) = 11.39, *p* < 0.001, _p_ω^2^ = 0.09. Follow-up pairwise comparisons show that intention to buy products produced by sexual crossing (*M* = 4.63, *SD* = 1.25) was significantly stronger than for products produced by mutagenesis (*M* = 4.40, *SD* = 1.51, *p* = 0.002), GM (*M* = 4.20, *SD* = 1.49, *p* = 0.05), and CRISPR (*M* = 4.36, *SD* = 1.41, *p* < 0.001). H2a was therefore supported. H2b was only partially supported as whereas intention in relation to GM was statistically similar to mutagenesis (*p* = 0.281) and CRISPR (*p* = 0.881), mutagenesis differed significantly from CRISPR (*p* = 0.017, see Figure 1B).

### Risk T1

The within-subjects test showed a medium and significant effect of technology, *F*(3, 324) = 12.68, *p* < 0.001, _p_ω^2^ = 0.10. Pairwise comparisons show that risk perception of sexual crossing (*M* = 3.93, *SD* = 1.25) was significantly lower compared to risk attributed to mutagenesis (*M* = 4.57, *SD* = 1.19, *p* < 0.001), and GM (*M* = 4.33, *SD* = 1.33, *p* = 0.004), but not statistically different from CRISPR (*M* = 4.00, *SD* = 1.33, *p* > 0.09), providing partial support for H3a. H3b was also only partially supported as perception of risk was not similar across mutagenesis, GM and CRISPR. Specifically, whereas risk perception did not differ significantly between GM and CRISPR (*p* = 0.065) and GM and mutagenesis (*p* = 0.177), mutagenesis was perceived as riskier than CRISPR (*p* < 0.001; see Figure 1C).

Although not hypothesized, we also computed a total score for each technology. We reasoned that more positive attitudes should be associated with lower risk and higher intention to buy. We therefore added the scores on attitude and intention to buy and subtracted the sum from perception of risk [(A + I) – R]. The higher the score, the more positively the technology was perceived. We then computed a repeated measures ANOVA comparing this total score among the four technologies. The assumption of sphericity was met. The results showed a large effect of technology, *F*(3, 324) = 17.90, *p* < 0.001, _p_ω^2^ = 0.13. Pairwise comparisons show that the composite score for sexual crossing (*M* = 5.54, *SD* = 2.94) was significantly more positive than for GM (*M* = 4.54, *SD* = 3.28, *p* = 0.002) and mutagenesis (*M* = 3.65, *SD* = 3.12, *p* < 0.001) but not for CRISPR (*M* = 5.18, *SD* = 3.13, *p* = 0.808). Moreover, the composite score for mutagenesis was significantly lower than those for GM (*p* = 0.017), and CRISPR (*p* < 0.001) while GM was not statistically different from CRISPR (*p* = 0.131, see Figure 1D).

### Knowledge of How Food Is Developed

**FIGURE 2:**
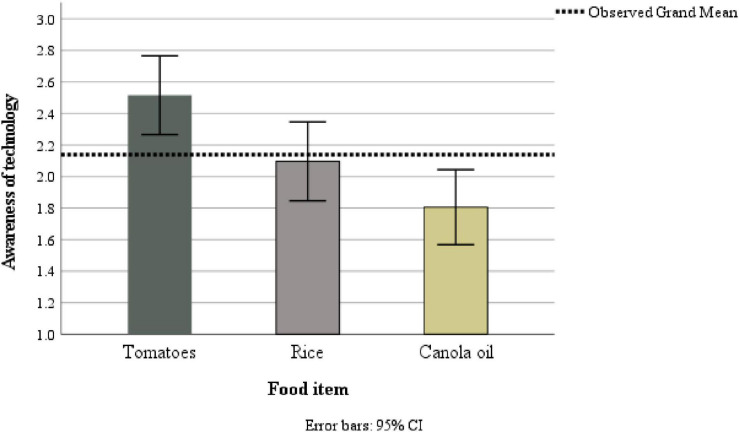
Awareness of how the different food items could be developed.

We tested whether there were any differences in knowledge of how the three presented foods could have been bred based on participants’ responses at T2. To this end we computed a repeated measures ANOVA. The Mauchly’s test of sphericity was not significant so sphericity was assumed. The within-subjects test showed a large significant effect of technology, *F*(2, 184) = 16.98, *p* < 0.001, _p_ω^2^ = 0.15. As illustrated in Figure 2, knowledge was highest for tomatoes (i.e., sexual crossing) and lowest for canola (GM). Pairwise comparisons show that participants had significantly more knowledge that tomatoes (*M* = 2.52, *SD* = 1.21) are developed by sexual crossing than that rice (*M* = 2.10, *SD* = 1.22) is developed by radiation (*p* = 0.002) and canola oil (*M* = 1.81, *SD* = 1.15) by GM (*p* < 0.001). Participants also reported marginally more knowledge about how rice is developed compared to canola (*p* = 0.052).

We also conducted a series of one sample *t*-tests to examine whether knowledge of the three technologies was significantly different from the midpoint (i.e., 3 = moderate amount of knowledge). All comparisons were statistically significant, with large effect sizes, showing that knowledge of all technologies was below the midpoint and closer to little than to moderate (sexual crossing: *t*[92] = −3.85, *p* < 0.001, *d* = 1.21; mutagenesis: *t*[92] = −9.97, *p* < 0.001, *d* = 1.15; GM: *t*[92] = −7.16, *p* < 0.001, *d* = 1.22). In line with H4, knowledge of breeding technologies was generally low, but lowest for mutagenesis and GM (i.e., rice, canola oil).

### Change From T1 to T2

To test whether attitudes, intention to purchase and risk perception changed from T1 to T2 (H5a, H5b, H5c) we conducted a series of one-tailed paired samples *t*-tests. The results displayed in Table 1 show that significant increases in attitudes (H5a) occurred only for mutagenesis and GM; significant increases in intention (H5b) to buy occurred only for mutagenesis; and significant decreases in risk (H5c) occurred only for mutagenesis and GM. The effect sizes were generally small. As such these hypotheses were partially supported as for sexual crossing there were no increases in attitudes and intention to buy, and no decrease in risk perception. For GM there was no significant change in intention to buy. On the other hand, H5d was supported as no changes occurred for CRISPR.

**TABLE 1:** One-tailed *t*-tests comparing the means (SDs within parentheses) for attitudes, risk, and intention at Time 1 and Time 2.

Technology	Variable	Time 1	Time 2	*t*(91)	Mean difference	95% CI	*p*	Cohen’s *d*
Crossing	Attitudes	4.84 (1.11)	4.93 (1.09)	–0.59	–0.06	[−0.27, 0.15]	0.278	0.09
	Risk	3.93 (1.25)	3.83 (1.22)	1.27	0.16	[−0.09, 0.40]	0.103	0.09
	Intention to buy	4.63 (1.25)	4.85 (1.34)	–1.44	–0.17	[−0.41, 0.07]	0.077	0.19
Mutagenesis	Attitudes	4.28 (1.11)	4.56 (1.25)	–2.22	–0.24	[−0.45, -0.02]	**0.015**	0.27
	Risk	4.57 (1.19)	4.32 (1.34)	1.77	0.22	[−0.03, 0.48]	**0.040**	0.21
	Intention to buy	3.94 (1.51)	4.40 (1.50)	–2.41	–0.39	[−0.72, -0.07]	**0.009**	0.30
GM	Attitudes	4.67 (1.12)	4.86 (1.20)	–1.86	–0.19	[−0.39, 0.01]	**0.033**	0.20
	Risk	4.33 (1.33)	4.10 (1.28)	1.94	0.24	[−0.01, 0.48]	**0.028**	0.20
	Intention to buy	4.20 (1.49)	4.44 (1.58)	–1.22	–0.18	[−0.47, 0.11]	0.114	0.17
CRISPR	Attitude	4.82 (1.16)	4.81 (1.18)	0.54	0.06	[−0.16, 0.29]	0.295	0.01
	Risk	4.00 (1.33)	4.01 (1.33)	–0.20	–0.03	[−0.31, 0.26]	0.420	0.01
	Intention to buy	4.36 (1.41)	4.52 (1.48)	–0.62	–0.08	[−0.35, 0.18]	0.268	0.13

*CI = Confidence interval for the mean difference; *p* = Statistical probability. Values in bold are statistically significant at *p* < 0.05. Cohen’s *d* = effect size.*

### Are Changes Between T1 and T2 Associated With Attitude-Behavior Discrepancy?

To be able to test whether the magnitude of change from T1 to T2 was associated with attitude-behavior discrepancy (i.e., discrepancy in scores between consumption behavior and attitude/intention/risk associated with each technology), discrepancy scores were calculated by subtracting the behavior score from the attitude/intention/risk score. Difference scores between T1 and T2 were calculated by subtracting the T2 score from the T1 score for attitude/intention/risk. For each technology, a series of partial correlation analyses was then conducted between the discrepancy scores and the difference scores, controlling for liking of the respective food item. The correlations in Table 2 show that, with the exception of attitudes toward mutagenesis, all coefficients were statistically significant, indicating that higher discrepancy scores between behavior and attitude/intention/risk, were positively associated with greater change from T1 to T2.

**TABLE 2:** Means, standard deviations, and partial correlations, controlling for liking of the respective food item (i.e., Tomatoes, Rice, and Canola oil), between the discrepancy scores and difference scores.

	Sexual crossing	Mutagenesis	GM
	
Discrepancy with behavior	Difference T1–T2		
Attitudes	0.39***	0.16	0.29**
Intention	0.38***	0.50**	0.50**
Risk	0.46***	0.40***	0.40***

**p < 0.01 and ***p < 0.001.

### Differences in Likelihood of Purchasing Specific Food Items

**FIGURE 3:**
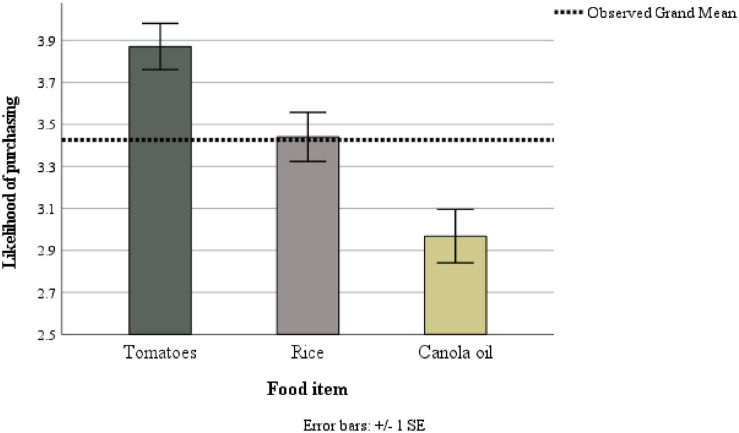
Means (SDs in Parentheses) for the intention to purchase the three presented foods bred through different technologies. Results of Greenhouse-Geisser corrected test.

To test whether participants had similar intentions to buy specific food items (i.e., tomatoes, rice, canola) bred through the respective technology, a repeated measures ANOVA was conducted. The within-subjects test was significant and with a large effect size, *F*(1.80, 165.81) = 21.91, *p* < 0.001, _p_ω^2^ = 0.18. Pairwise comparisons show that participants were significantly more likely to report that they would buy tomatoes (*M* = 3.87, *SD* = 1.07) compared to rice (*M* = 3.44, *SD* = 1.13, *p* = 0.008) and canola (*M* = 2.97, *SD* = 1.23, *p* < 0.001). Participants were also significantly more likely to buy rice compared to canola (*p* < 0.001; see Figure 3).

## Discussion

There has been widespread community concern around the use of GM technology in plant breeding since the 1990s (Blancke et al., 2015). This concern and the government regulation that has followed has limited its application in plant breeding programs. The recent development of CRISPR technology has once again presented researchers and breeders with both a potentially valuable tool and a challenge in terms of gaining a social license for its use. In contrast to GM, mutation breeding has been used for the development of new crop varieties since the 1930s but has faced little to no community concern and government control. We set out to test whether different biotechnologies (i.e., sexual crossing, mutagenesis, GM, CRISPR) are associated with contrasting attitudes, intentions to buy and perceived risks. We expected attitudes (H1a) and intentions (H2a) to sexual crossing to be more positive than attitudes and intentions to all of the other technologies presented, and risk perception to be lower (H3a). These expectations were met only for intentions (H2a). Attitudes to sexual crossing were only more positive than attitudes to mutagenesis; and perceived risk was only lower than the risk perceived for mutagenesis and GM but not CRISPR. On the other hand, we expected that at a baseline there would be no differences in attitudes, intention and risk perception between mutagenesis, GM, and CRISPR (H1b, H2b, H3b). These hypotheses were only partially supported. Specifically, attitudes were similar between GM and CRISPR, but these two technologies differed from mutagenesis. There was similarity in relation to intention between mutagenesis and GM, whereas CRISPR differed from mutagenesis. Risk perception differed between CRISPR and mutagenesis, but these two technologies did not differ from GM.

In terms of changes to attitudes, intentions, and risk perception from T1 to T2, where participants learned how specific foods were developed, three of the four hypotheses were partially supported. Specifically, attitudes became more positive (H5a) only for mutagenesis and GM. Intention became stronger (H5b) only for mutagenesis, and perceived risk decreased (H5c) only for mutagenesis and GM. On the other hand, H5d was supported as scores for CRISPR stayed the same over time. In addition, H6 was supported as there were significant positive associations between discrepancy scores and difference scores across all technologies, indicating cognitive dissonance and that the discomfort provoked by the attitude-behavior inconsistency was resolved by changing the attitude (i.e., attitude, intention and risk perception).

### Differences Between Attitudes, Intention, and Risk Perception

Whereas not all of the results were in line with expectations, they shed interesting light on the plant breeding technology debate. Although plant breeding through induced mutagenesis has been considered safe (European Commission, 2018), when participants received factual information about this technology, their attitudes were more negative than for all other technologies. They associated it with the greatest risk, with the exception of the use of GM technology. A likely explanation for this is a general lack of knowledge about the technologies (see McPhetres et al., 2019) and the tendency to see the terms mutagenesis and radiation negatively. In line with H4, our results have shown that knowledge about plant breeding was generally low. Research shows that when people are uncertain about their knowledge, they tend to rely on evaluations and opinions of others they believe are more knowledgeable and are considered trustworthy (Deutsch and Gerard, 1955; Williamson et al., 2013). In the case of GM foods, opinion building via NGOs such as Greenpeace has likely either directly or indirectly guided the formation of the negative opinions displayed by the participants. It may have also induced the public into believing that they have more knowledge of the issue than they really do (Fernbach et al., 2019). Because a debate about food produced by induced mutagenesis has been lacking, this kind of informational influence is not available. As such, participants relied on the information we provided them, which contained language with connotations to radioactivity, which is likely to be associated with pre-formed concerns around health. Research into consumer attitudes to post-harvest food irradiation (e.g., Evans and Cox, 2006; Behrens et al., 2009; Caputo, 2020) has shown that attitudes to the use of this technology tend to be negative. Although there are important, fundamental differences between radiation induced mutagenesis in plant breeding (use in progenitors) and post-harvest food irradiation (direct usage on produce consumed), this suggests that terminology may frame information in ways that impact the perception of an issue.

Attitudes and risk perception did not differ significantly in relation to sexual crossing and gene editing (CRISPR). It is understandable that attitudes to sexual crossing were positive and risk perception was low, as this technology is readily associated with natural processes. Gene editing, on the other hand, may well have been expected to be associated with GM. Indeed, to some extent, this seems to have been the case, as attitudes, intention and risk perception did not differ significantly between the two technologies. However, scores on these variables were more positive for gene editing than for GM. Gene editing is a still relatively unknown technology that has so far largely escaped public debate and, thus, the negative publicity that GM has attracted (but see Cotter and Perls, 2018). The labels “gene editing” or “CRISPR” are likely not imbued with negative content, and participants’ opinions, therefore, could be expected to be relatively free from pre-existing biases.

### Changes as a Function of Knowledge

The results show that whereas attitudes, intention and risk perception did not change in relation to sexual crossing, once participants had learned about foods developed through each of the technologies (CRISPR excluded), they changed their attitude in relation to mutagenesis and GM (except intention). Specifically, the participants’ scores on attitudes and intention toward mutagenesis increased while risk perception decreased. A similar pattern was shown for GM with regards to attitudes and risk perception. These changes were likely due to arousal of cognitive dissonance as a result of knowing that one’s attitudes were not in line with one’s behavior (Festinger, 1957; Festinger and Carlsmith, 1959). That is, when participants realized that they were probably eating foods developed through technologies that they disapproved of, or believed are associated with risk, their attitude and perception changed in order to alleviate psychological discomfort (Festinger, 1957). The correlations between discrepancy and difference scores support this interpretation. The lack of change in relation to sexual crossing and CRISPR also supports this explanation as these technologies were unlikely to cause cognitive dissonance. Sexual crossing is likely to be perceived as a natural process and as such there is no inconsistency between attitude and behavior and, therefore, there was no need for participants to change their perceptions. With CRISPR, because no foods that participants could already be consuming were presented, there was no inconsistency and, consequently, no need for change.

### Likelihood of Buying Plant Foods Bred Through Sexual Crossing, Mutagenesis, and GM

We tested the likelihood of participants buying food items developed through each of the technologies (except CRISPR). The participants were most likely to purchase a food item developed through sexual crossing (tomatoes) followed by mutagenesis (rice) and GM (canola oil). Although it somewhat contradicts the more general intentions tested in H2a, as well as the general tendency to rate mutagenesis more negatively overall (i.e., attitude and risk), this could be due to the produce that were associated with the technologies in the fact sheets and pictures (i.e., wheat and rice for mutagenesis, and canola oil for GM). Whereas both wheat and rice are staple crops that people are likely to consume and are harder to substitute, canola oil is not a staple and can be substituted by other types of oil. As such, it is unclear whether the stronger intentions to purchase foods developed through mutagenesis than through GM was caused by the technology or the produce.

## Implications

We argue that this research illustrates the inconsistencies inherent in the debate and regulation of plant breeding technologies. In a time of strong population growth, diminishing availability of productive agricultural land, and changing growing conditions due to climate change, it is important that science contributes to improving agriculture and food security. Governments make decisions on which plant breeding methods are acceptable, and government decisions are informed by public opinion (European Commission, n.d.; Wolt and Wolf, 2018) and not exclusively on scientific evidence. For this reason, NGO’s messaging about different technologies can impact government decisions by mobilizing public opinion. The concern is that governments are being informed by positions that may, ultimately, be detrimental to society (Agre et al., 2016). Our findings on mutagenesis highlight the core of the problem by showing the inconsistencies surrounding plant breeding regulation. That is, not all plant breeding technologies undergo similar scrutiny. One possible implication of our findings is that if public opinion is to inform decision making, then induced mutagenesis could, by extension, be subject to the same strict regulations as GM. Legislators have, however, broadly accepted that plants with induced mutations are safe (European Commission, 2018). Rather than suggesting strict regulations on mutagenesis, similar to those applied to GM, we argue that our findings make the case for a rethinking of the decision-making process of plant breeding, where scientific evidence, rather than opinion, is prioritized and a consistent approach is applied.

## Limitations

Although this research presents findings that show inconsistencies between public attitudes to plant breeding technologies and government regulation of those technologies, there are some methodological limitations that need addressing. The scales used to measure attitudes, intention, and risk were not ideal because the questions had qualifications attached to them (e.g., “Food produced with this technology will be useful for the *fight against third world hunger*”). The wording “fight against third world hunger” adds a positive tone to the statement, which could have induced a more positive evaluation of the technology. Future research should therefore use more neutral scales to assess these variables. The pictures of foods shown to participants at T2 could also have introduced confounds that could not be controlled. Different foods are likely to have different degrees of attractiveness or liking. Rice, tomatoes, and canola oil are qualitatively different. Whereas rice is a staple food and essential in many cultures, tomatoes are more of an added ingredient to cooking, and canola oil is mostly used to cook other foods and can be substituted for other oils. As such these food items were not equivalent and may have affected the results in unknown ways. Because we aimed to be true to reality and, in Australia only canola oil is commercialized for human consumption, we were limited in relation to which GM foods we could present. However, future research should aim to select foods presented controlling for various parameters to rule out confounds (e.g., familiarity, arousal etc.) as done in food perception research (Foroni et al., 2016; Coricelli et al., 2019; Mengotti et al., 2019) or to calibrate them in terms of such parameters.

The sample also imposes some limitations with regards to generalizability as it consists of a convenience sample of university students. It could be argued that university students are more knowledgeable of science than the public in general and, consequently, have greater knowledge of plant food biotechnology. Although, we did not ask participants about their general knowledge of such technologies, we asked about their knowledge about how specific food items were developed (i.e., tomoatoes, rice, canola). The levels of knowledge were generally low, suggesting that any differences between our sample and the general population are likely to be minimal. It could also be argued that university students have greater ability to process complex information compared to the general public, which could have skewed the results. Although, research on similar issues from a variety of countries (e.g., Evans and Cox, 2006; Behrens et al., 2009; Caputo, 2020) suggests that the current results would be replicated in other jurisdictions and samples we caution against making broader generalizations without further replication in other more representative samples.

Arguably the change from T1 to T2 could also be only temporary and simply an immediate response to a situation of psychological discomfort. It could be that, once equipped with the new knowledge, in the long run, participants would change their behavior rather than their attitude. For example, they could become choosier when making purchases of food items to make sure that they consume goods that are in line with their ideological position. However, attitude change is determined by many factors such as the source and quality of the message, and the recipient’s motivation (Crano and Prislin, 2006). These factors were not measured, which prevent us from drawing conclusions with regards to the likelihood of long-term change. Alternatively, a third measurement conducted, perhaps 6 months after T2, could indicate whether any changes were lasting. In the absence of this knowledge, all that can be concluded is that the information received led to an immediate change in attitude. Additionally, although a within-subjects design with baseline measures, where participants serve as their own control, provides a measure of control for the effect of independent variables, the use of a control group that did not get any information about plant breeding technology could be used in future research to lend stronger conclusions.

It could also be argued that our results reflect the way the information was framed. Whereas we attempted to frame the information as neutral and scientifically factual, the fact that we did not convey any negative aspects potentially associated with plant biotechnology (e.g., vested interests of multinationals, or potential patentability of life forms), could be viewed as influencing participants in a positive direction. In hindsight, the content about the outcomes of each technology (e.g., disease resistance, larger yield), could have been perceived as positive. On the other hand, these outcomes could also be perceived as unnatural (i.e., negative) alterations to the original plant with all the associations this may have had for participants. The current data do not provide enough information to clearly unpack the issue of positivity and negativity. However, the positive position of gene editing in the measured variables relative to GM and mutagenesis helps to disentangle it somewhat. Due to its relative novelty in the public mind, it is unlikely to be strongly associated with any narrative (positive or negative), the way GM is likely to be. Its label (i.e., CRISPR/gene editing) is also unlikely to conjure up risks in the way that radiation (i.e., mutagenesis) is likely to. This lack of positive/negative history and connotations associated with gene editing, makes it possible to use is as a kind of control. If the framing of the information influenced participants in a positive direction, this influence should have had a uniform effect across technologies, both in terms of perception as well as change. The results, in particular for gene editing, compared to GM and mutagenesis suggest that label connotations (mutagenesis) and historical (negative) narrative (GM) suppressed positive perception and change.

## Conclusion

This study aimed to investigate attitudes, intentions, and risk perceptions toward multiple plant breeding technologies and to examine whether scores on these variables would change once the participants received more information about foods developed through the use of the different technologies. The results showed that, once participants learn the characteristics of the different techniques, mutagenesis stood out as the technology with the least positive evaluations. It also showed, importantly, that participants tended to change their evaluations once they learned more about foods developed through the different methods that are already commercialized. Taken together, the results suggest a re-examination of the basis of the decisions that underly the regulation of plant breeding technologies. The underpinning importance of breeding to agriculture and by extension the global environment and our societies warrants a rigorous, considered, and consistent approach.

## Data Availability Statement

The datasets presented in this study can be found in online repositories. The names of the repository/repositories and accession number(s) can be found below: Figshare 10.6084/m9.figshare.13624202.

## Ethics Statement

The studies involving human participants were reviewed and approved by the Ethics Committee, Australian Catholic University. The participants provided their written informed consent to participate in this study.

## Author Contributions

LB planned the study, collected the data, and wrote the manuscript. FF and BJ contributed equally with ideas for the planning, the data analyses, and the writing of the manuscript. All authors contributed to the article and approved the submitted version.

## Conflict of Interest

The authors declare that the research was conducted in the absence of any commercial or financial relationships that could be construed as a potential conflict of interest.

## Footnotes

1 https://www.acu.edu.au/research/research-ethics-integrity-and-compliance/research-ethics

2 https://doi.org/10.6084/m9.figshare.13624202.v1

## Appendix A

### Attitudes to Breeding Technology

Food produced with this technology will be useful for the fight against third world hunger.

1. In the long run, a food industry using this technology will be good for the Australian economy.
2. Whatever the dangers of this food technology, future research will deal with them successfully.

### Perceived Risks

1. Eating food developed through this technology will be harmful to my health and my family’s health.
2. This food technology threatens the natural order of things.
3. Growing crops with this technology will be harmful to the environment.

### Consumer Intentions

1. I would buy food developed through this technology if it contained less fat than ordinary food.
2. I would buy food developed through this technology if it were cheaper than ordinary food.
3. I would buy food developed through this technology if it were grown in a more environmentally way.

## Appendix B

### Introduction to Breeding Technologies

“Plant breeding is the science of changing the genes and traits of plants in order to produce desired characteristics. It has been used to improve the quality of nutrition in products for humans and animals. Plant breeding can be accomplished through many different techniques ranging from simply selecting plants with desirable characteristics for propagation, to methods that make use of knowledge of genetics and chromosomes, to more complex molecular techniques. Genes in a plant are what determine what type of qualitative or quantitative traits it will have. Plant breeders strive to create a specific outcome of plants and potentially new plant varieties.

Plant breeding has been practiced for thousands of years, since near the beginning of human civilization. It is practiced worldwide by individuals such as gardeners and farmers, and by professional plant breeders employed by organizations such as government institutions, universities, crop-specific industry associations or research centers. Some qualities breeders aim to develop are, for example, better tolerance to drought or resistance to newly evolved viral or bacterial disease, greater crop yield or stronger flavor.

### Breeding Technologies Fact Sheets

#### Sexual Crossing (Classical Plant Breeding)

Classical plant breeding uses deliberate interbreeding (crossing) of closely or distantly related ‘individuals’ to produce new crop varieties or lines with desirable properties. Plants are crossbred to introduce traits/genes from one variety or line into a new genetic background. Breeders cross the pollen from one plant with a desired quality with another plant. For example, a mildew-resistant pea may be crossed with a high-yielding but susceptible pea, the goal of the cross being to introduce mildew resistance without losing the high-yield characteristics. Progeny from the cross would then be crossed with the high-yielding parent to ensure that the progeny was most like the high-yielding parent (backcrossing). The progeny from this cross would then be tested for yield and mildew resistance and high-yielding resistant plants would be further developed. The series of multiple backcrosses to eliminate the undesirable parent DNA can take years to perform.

#### Mutagenesis

Involves the process of exposing seeds to chemicals or atomic radiation in order to generate mutants with desirable traits to be bred with other cultivars. Plants created using mutagenesis are sometimes called mutagenic plants or mutagenic seeds. From 1930 to 2014 more than 3200 mutagenic plant varieties were released that have been derived either as direct mutants or from their progeny. This method is used, for example, when a novel disease appears that no plants have resistance to. Through this method it is possible to randomly mutate DNA in parental plant material in order to shuffle the DNA so that a novel random DNA combination that has resistance might be identified. Plants can be mutated by chemical or gamma radiation mutagenesis. Both of these induce mutations by randomly breaking apart the DNA so that it comes back together in novel arrangements. It can be described as accelerated evolution of DNA. Wheat is a typical example of a mutagenesis crop.

#### GM

These are plants where the DNA has been modified using genetic engineering methods. In most cases, the aim is to introduce a new trait to the plant which does not occur naturally in the species. Examples in food crops include resistance to certain pests, diseases, environmental conditions, reduction of spoilage, resistance to chemical treatments (e.g., resistance to a herbicide), or improving the nutrient profile of the crop. If DNA that gives resistance to a disease is not present in a parent plant, it is possible to take the DNA from virtually any organism and insert it into a production plant. A typical example of this is insect resistant cotton plants that contain DNA from a bacterium that produces a toxin that kills the insect when it consumes the bacteria. Inclusion of this bacterial DNA fragment in cotton plants enables them to produce the toxin that kills the insect pests when the cotton leaves are consumed by the insect. Canola oil is a typical example of a GM crop.

#### Gene Editing (CRISPR)

This is a new technology that allows the precise correction of gene fragments. This technique can be used for various purposes: from the improvement of crops to make them disease-resistant, or to improve yield and nutritional quality. This technique can take several forms but basically it allows breeders to target a particular DNA sequence so that it can be changed to introduce a novel characteristic. In contrast to some of the other techniques, gene or base editing is targeted rather than random and is designed to rewrite a plant’s own DNA rather than introducing DNA from another plant or organism to introduce a novel characteristic. The ability to precisely rewrite a plant’s own DNA to gain a desired characteristic can accelerate the breeding process. An example of this technique is non-browning button mushrooms which have had the amount of enzyme that causes them to turn brown reduced. The end-product is a mushroom with longer shelf life that resists blemishes caused by handling or mechanical harvesting.
